# Improving physical activity and screen time in Australian Outside School Hours Care: Study protocol

**DOI:** 10.1038/s41390-024-03464-1

**Published:** 2024-08-23

**Authors:** Carol Maher, Hayley Christian, Nicole Nathan, Anthony Okely, Svetlana Bogomolova, Lucy K. Lewis, Dylan P. Cliff, Adrian Esterman, Rachel Milte, Richard R. Rosenkranz, Rachel G. Curtis, Jacinta Brinsley, Ty Ferguson, Rosa Virgara, Mandy Richardson, Kylie Brannelly, Rebecca Stanley, Natasha Schranz, Perry Campbell, R. Glenn Weaver, Michael Noetel, Luke Wolfenden

**Affiliations:** 1 Alliance for Research in Exercise, Nutrition and Activity (ARENA); University of South Australia, Adelaide, SA Australia; 2 Telethon Kids Institute, Perth, WA Australia; 3 School of Medicine and Public Health; The University of Newcastle, Newcastle, NSW Australia; 4 School of Health and Society; University of Wollongong, Wollongong, NSW Australia; 5 Centre for Social Impact; Flinders University, Adelaide, SA Australia; 6 Caring Futures Institute; Flinders University, Adelaide, SA Australia; 7 Early Start, School of Education; University of Wollongong, Wollongong, NSW Australia; 8 Department of Kinesiology; Kansas State University, Manhattan, KA USA; 9 Department of Kinesiology and Nutrition Sciences; University of Nevada, Las Vegas, NA USA; 10 Department for Education, Adelaide, SA Australia; 11 Queensland Children’s Activities Network, Woodend, QLD Australia; 12 Wellbeing SA, Adelaide, SA Australia; 13 Australian Children’s Education and Care Quality Authority (ACECQA), Darlinghurst, NSW Australia; 14 Department of Exercise Science, Arnold School of Public Health; University of South Carolina, Columbia, SC USA; 15 School of Psychology; The University of Queensland, St Lucia, QLD Australia

## Abstract

**Background:**

Children’s physical activity and screen time behaviours impact their physical health and well-being. In Australia, less than half of children meet daily physical activity recommendations and only one-third meet daily screen time recommendations. Nearly half a million Australian school children aged 5-12 attend Outside School Hours Care (OSHC) weekly, activities undertaken at OSHC play a key role in meeting these recommendations. Currently, physical activity and screen time practices in OSHC vary and lack policy guidance. The Activated OSHC program is a policy-based intervention that supports OSHC services to implement the physical activity and screen time guidelines.

**Methods:**

192 OSHC services across Australia will be recruited. 96 services will be randomly allocated to receive the Activated OSHC program. OSHC coordinators will complete online surveys examining physical activity and screen time scheduling, cost, acceptability, and feasibility. Primary outcome; changes in the proportion of intervention and control services meeting OSHC sector physical activity and screen time guidelines, and secondary outcomes; changes in children’s physical activity and screen time behaviours; changes in staff behaviour will be assessed using mixed-effects regression models.

**Discussion:**

The aim of this study is to examine the impact of the Activated OSHC program on children’s physical activity and screen time.

**Impact:**

- Recent Australian research in Outside School Hours Care (OSHC) has identified significant inconsistency in practices related to physical activity and screen time, compounded by an absence of explicit policy guidance.
- The Activated OSHC program is a policy-based intervention that supports OSHC services to implement the Australian OSHC physical activity and screen time guidelines.
- This study will assess the implementation and effectiveness of the Activated OSHC program in an effectiveness-implementation hybrid type 2 trial design.
- Implementation of outside school hours care sector physical activity and screen time guidelines may improve children’s physical activity and screen time behaviours.

## Introduction

Children’s lifestyle behaviours, including their daily physical activity and screen time behaviour, impact their physical health, psychosocial well-being, and academic performance.^1^ In Australia, evidence shows that only a minority (15-40%) of children meet the physical activity guideline of 60 minutes per day at moderate-to-vigorous intensity, and only about one-third (32%) adhere to the screen time guideline of less than 2 hours per day,^2^ underscoring the need for novel strategies to address these behavioural patterns. Particularly, the need for scalable, low-cost cost, and easily implementable solutions that are contextually appropriate for the environments where children spend their time is paramount.

Substantial efforts have focussed on designing interventions targeting children’s physical activity and screen time in school.^3^ and family.^4^ contexts. Relatively less attention has been paid to childcare settings, particularly those serving elementary school-aged children (i.e., children aged approximately 5-12 years). Around the globe, millions of children attend formal childcare services before or after school to fit their parents’ work schedules, engage in enrichment activities, foster social interactions, and receive homework support.^5–7^ In the United States, such services are typically known as “After-School Programs”, while in Australia, they are referred to as Outside School Hours Care (OSHC). Approximately 10% of Australian school children between the ages of 5 and 12 participate in OSHC every week.^5^ The duration of their stay at OSHC is considerable, with before-school care sessions lasting up to approximately 2 hours, after-school care approximately 3.5 hours, and vacation care extending to approximately 11 hours per day. Consequently, the types of activities facilitated in OSHC have an impact on whether children meet daily physical activity and screen time guidelines.

Recent Australian research in OSHC has identified significant inconsistency in practices related to physical activity and screen time, compounded by an absence of explicit policy guidance.^8^ Observational studies carried out in randomly selected OSHC services in South Australia (SA).^8^ and New South Wales (NSW).^9^ found a broad range in recreational screen time practices, from offering none through to 5.5 hours per day in different OSHC services. Similarly, physical activity opportunities varied substantially, with some facilities failing to offer daily physical activities and others designating several hours for active play.^8^ In addition, a recent survey revealed that only 16% of OSHC coordinators were familiar with the Australian government’s 24-hour physical activity and screen time guidelines.^10^

**Fig. 1:**
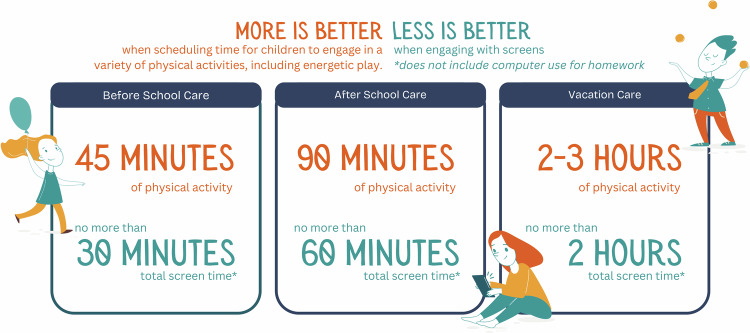
The Australian OSHC sector physical activity and screen time guidelines.

Throughout 2019-20, we led a stakeholder consultation process to develop evidence-based physical activity and screen time guidelines for the Australian OSHC sector.^11^ The work was informed by a scoping review of OSHC-related physical activity and/or screen time guidelines around the world,^12^ a systematic review of OSHC-related physical activity intervention studies,^13^ and an international Delphi study which surveyed 67 stakeholders, including OSHC staff, families, government representatives, and child health experts.^11^ During the four-round Delphi process, we developed the first Australian OSHC physical activity and screen time guidelines; the first national scale OSHC guidelines anywhere in the world. The draft guidelines were provided to all Australian OSHC coordinators (*n* = 3518) for feedback, with 566 (16%) responding.^10^ The final guidelines (Fig. 1) use a 3:1 ratio for offered physical activity to physical activity achieved, based on the rationale that children are not physically active for the entire duration of playtime. Consequently, the guidelines recommend that OSHC services offer 45 minutes of active play before school, 90 minutes after school, and 180 minutes during vacation care sessions. These durations are designed to result in children achieving 15 minutes, 30 minutes, and 1 hour of physical activity, respectively. These recommendations align with the Australian physical activity guidelines, which suggest that children should accumulate at least 60 minutes of MVPA each day.^14^ By offering physical activity within OSHC, these guidelines help ensure that children have ample opportunities to meet their daily physical activity requirements. The guidelines have been endorsed by the national peak body, the Australian National Outside School Hours Services Alliance (NOSHSA).^15^

While the development of guidelines is a critical first step towards improving physical activity and screen time practices in an OSHC setting, their creation alone will not instigate changes in practice. Behaviour change interventions are required to implement the physical activity and screen time guidelines within services across the OSHC sector. The Activated OSHC program is a policy-based intervention that supports OSHC services to implement the Australian OSHC physical activity and screen time guidelines. The current study will assess the implementation and effectiveness of the Activated OSHC program in an effectiveness-implementation hybrid type 2 trial design.

This study has five research questions:

1. What is the effectiveness of the Activated OSHC program for increasing the proportion of OSHC services meeting the new evidence-based Australian OSHC sector physical activity and screen time guidelines? (implementation outcome)
2. What is the effectiveness of the Activated OSHC program for *increasing* the proportion of time children spend being physically active at OSHC? (effectiveness outcome)
3. What is the effectiveness of the Activated OSHC program for *reducing* the proportion of time children spend in recreational screen time at OSHC? (effectiveness outcome)
4. What is the cost-effectiveness of the Activated OSHC program?
5. What is the acceptability and feasibility of the Activated OSHC program?

## Methods

### Study design

This study is a Type 2 hybrid effectiveness-implementation randomised controlled trial (RCT), with assessments at baseline, 3, and 12 months. The unit of randomisation and unit of analysis is at the OSHC service level. All participants will provide informed consent to participate. Ethical approval for the study was obtained from the Human Research Ethics Committee of the University of South Australia (protocol number: 204366) and the trial is registered with the Australian and New Zealand Clinical Trial Registry (registration number: ACTRN12622000393752). The conduct and reporting of the trial will adhere to the Consolidated Standards of Reporting Trials (CONSORT) guidelines and the intervention will be described according to the Template for Intervention Description and Replication (TIDieR) checklist.^16^ A rolling recruitment is expected to take place from August 2022 to December 2023, with final follow-up assessments expected to be completed by December 2024.

### Participants and recruitment

**Fig. 2:**
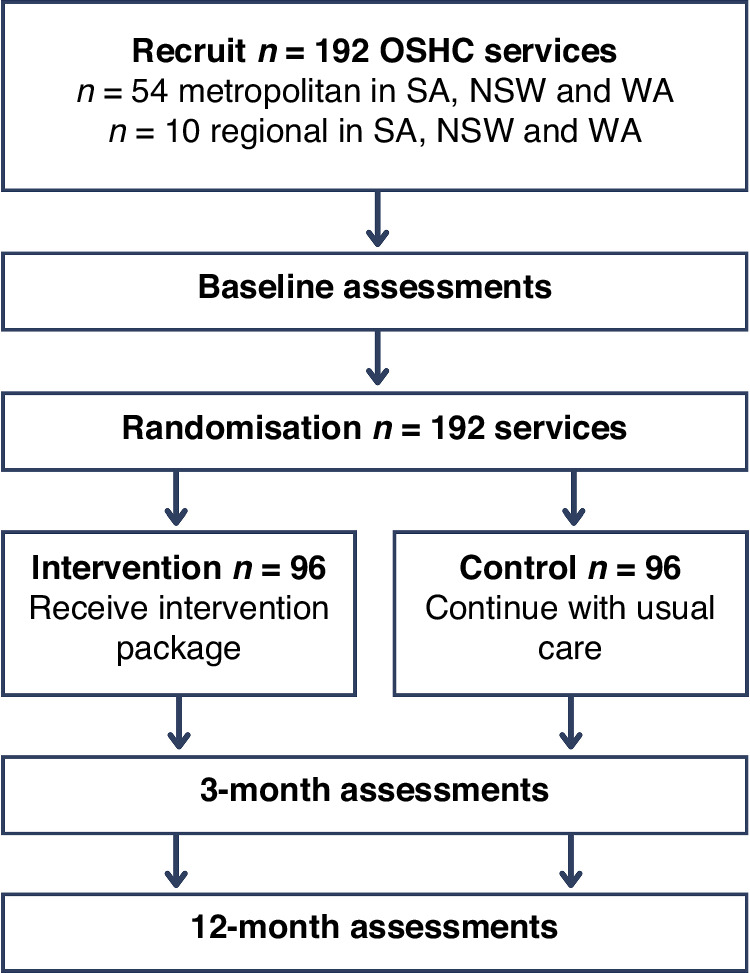
CONSORT participant flow diagram for recruitment.

Eligible OSHC services will be those that offer both before- and after-school care, for at least 10 children on a typical day, have internet access, and have an OSHC coordinator who is able to speak, read, and write in English. A list of eligible OSHC services within 120 km radii of Adelaide (in the state of South Australia), Newcastle (New South Wales), and Perth (Western Australia), will be obtained from the Australian Children’s Education and Care Quality Authority (ACECQA) and will serve as the sampling frame. To enhance the sample’s representativeness, services will be divided into socioeconomic status (SES) tertiles, based on their ‘Index of Community Socio-Educational Advantage’ (ICSEA) score. ICSEA is an Australian government metric that provides a standardised measure of the socio-educational backgrounds of students, taking into account various factors such as parents’ education levels, occupation, and location (urban/rural) among others.^17^ Metropolitan services will be invited in a randomly selected order (computer generated sequence), and with a recruitment goal of 54 services per state (18 low, 18 medium, and 18 high SES) totalling 162 metropolitan services (Fig. 2). OSHC coordinators will be emailed a study information package, followed by telephone contact to confirm eligibility. For logistical reasons, services within a 0-50 km radius will be approached first. If the quota is not met, we will extend recruitment to services within 50–75 km, 75–100 km, and then 100–120 km radii until we reach our target. We will also recruit 30 regional services across the three states using convenience sampling. Regional services will be invited within geographic clusters (so that data collection is feasible) until 10 services in each state (total = 30) are recruited.

### Intervention

Activated OSHC is a policy-based intervention, with intervention components provided to support OSHC services to become an accredited Activated OSHC service. It was developed following a series of online co-design workshops with OSHC coordinator. The intervention is modelled on other successful accreditation-based interventions; the Cancer Council of Australia’s SunSmart program,^18^ which supports sun-safe practices in schools, OSHC, and childcare settings, and the Alcohol and Drug Foundation’s Good Sport program.^19^ which supports healthy drug and alcohol culture in community sporting clubs. Accreditation will initially last for 3 years, after which time services will need to reapply to renew their accreditation.

**Fig. 3:**
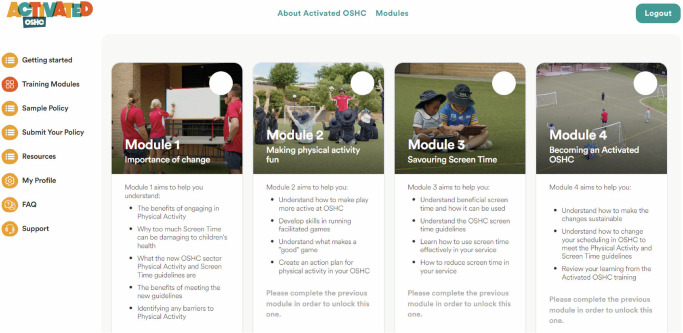
Image of the Activated OSHC training modules webpage.

The intervention components have been developed using the Theoretical Domains Framework (TDF) – an integrative implementation framework that synthesises behaviour change constructs that enable or pose barriers to the implementation of evidence-based practice.^20^ This framework has been widely used in effective clinical practice change interventions and has been previously applied by the research team to design implementation strategies to improve adherence to sector physical activity policy in schools.^21,22^ The implementation strategies are directly linked to enablers and barriers identified through extensive consultation with the OSHC sector and mapped to TDF constructs. Participants will be provided with the following resources to assist in implementing the physical activity and screentime guidelines:

- **“Becoming an Activated OSHC” manual** (Supplementary File 1): This manual provides education on the Australian OSHC physical activity and screen time guidelines, recommendations and examples of how to schedule physical activity and screen time, and practical tips to encourage active play. It also describes the process of completing the intervention to become an “Activated OSHC” (i.e., all staff to complete training modules, and develop an approved physical activity and screen time policy).
- **Online training resources**: A series of four online training modules will be provided (Fig. 3).

1. *Importance of Change* describes the benefits of becoming an “Activated OSHC”. It describes the physical activity and screentime guidelines, the benefits of physical activity for children, and the importance of staff role-modelling of healthy physical activity behaviours. It identifies and addresses barriers and enablers that OSHC educators face when trying to get children to engage in more physical activity.
2. *Making physical activity fun* describes how to make physical activity fun and engaging for children and how to increase physical activity scheduling, including practical tips on increasing children’s participation (e.g., avoiding lines or elimination games) and enabling children to choose how to be active (e.g., which games and how they’re played).
3. *Savouring screen time* describes how to reduce screen time (e.g., when to schedule screen time) and how to provide active screen-based games rather than passive screen activities. It also describes how to inform and involve families in the new scheduling changes.
4. *Becoming an Activated OSHC* examines staff knowledge of the OSHC physical activity and screen time guidelines, including scheduling, guideline limits, and ways to increase physical activity and reduce screen time.

c. **Posters**: Posters reminding staff, children, and families about the guidelines will be provided for display in OSHC services. These resources will help address key barriers and enablers identified in the national survey (e.g., potential resistance from the children to changing screen time practices).
d. **Family communication resources**: OSHC services will receive access to online resources (brochures plus newsletter-suited text) they can use to help families understand OSHC programming changes, and support integration into positive physical activity and screen time practices at home.
e. **Accredited Activated OSHC signage:** OSHC services that submit a physical activity and screentime policy that is assessed as meeting the OSHC Physical Activity and Screen time guidelines, and who have at least 50% of their staff complete the online training modules, will be accredited. They will receive signage to display at the OSHC service, and an email signature to promote their Accredited Activated OSHC status.

### Procedure

OSHC coordinators will provide informed consent for their service to take part in the study. They will then be given letters to send home to the parents and guardians of children at their service, providing information about the study and giving them the opportunity to ‘opt out’ of the study if they wish for their child to be excluded from observation. These children will wear an orange sticker on their clothing during observations to signify they will not be included in data collection.

Assessments will be conducted at baseline (prior to randomisation), 3 months, and 12 months post-baseline by research personnel blinded to group allocation. At each timepoint, OSHC coordinators will complete an online survey and their OSHC service will be visited by two research personnel to observe a before-school care session and an after-school care session. The visits will take place on an unannounced day to reduce observation bias, a method that has been used successfully in a previous study.^23^ The OSHC coordinator will be notified of a two-week window in which each assessment will occur but will not be told the exact day. In recognition of the time and effort of being involved, OSHC services that remain in the study at 12 months will be provided with a $250 honorarium to spend on sports equipment for their service, an active excursion, or staff professional development related to physical activity.

### Allocation and blinding

Following baseline data collection, OSHC services will be randomised by research staff in a 1:1 allocation ratio to either the intervention or control group. Randomisation will be stratified based on state and location (metropolitan or regional), with metropolitan services further stratified by SES tertile. Randomization will be undertaken using REDCap software. Block randomisation (3 states x 4 groups, i.e. regional or one of three metropolitan SES tertiles) will be used to ensure group allocation is equal.

Services randomised to the intervention group will receive access to the Activated OSHC program and will be encouraged to become an Accredited Activated OSHC service. Services randomised to the control group will be encouraged to continue with their usual programming and will receive access to the Activated OSHC program following completion of the 12-month assessment.

Participants cannot be blinded to condition, however, research staff conducting the assessment visits will be blinded to group allocation.

### Outcomes

The following implementation and effectiveness outcomes are planned:

#### Implementation outcome: Meeting the OSHC sector physical activity and screen time guidelines

Daily physical activity and screen time in before- and after-school care sessions will be assessed at each time point using the OSHC Schedule Tool to see if services meet OSHC physical activity and screen time guidelines (i.e. before-school physical activity ≥45 mins; before-school screen time ≤30 mins; after-school physical activity ≥90 mins; after-school screen time ≤60 mins). Completed by OSHC coordinators, this self-report tool logs daily activities in three categories: general (9 items), screen-based (7 items), and physical activities (7 items), with duration reported in 15-minute increments.^8^ The tool has acceptable validity when compared against directly observed levels of physical activity (*r* = 0.41) and screen time (*r* = 0.73).^8,10^

#### Effectiveness outcomes

##### Proportion of OSHC care sessions that children spend in moderate-to-vigorous physical activity and recreational screen time

Physical activity and screen time opportunities during a before-school and an after-school care session will be collected at each time point using the System for Observing Play and Leisure Activity in Youth (SOPLAY) direct observation method.^24^ The SOPLAY method involves two trained research personnel conducting continuous visual scans of children’s play zones for an entire care session. For each scan, the time, type of children’s activity, and the number of boys and girls engaged in sedentary behaviour (sitting or standing), light, or vigorous physical activity is recorded.^24,25^ This method has demonstrated acceptable reliability (IOA = 80%) and validity (*r* = 0.75).^26^ against accelerometer-measured physical activity. Interrater reliability will be captured through up to six reliability scans per hour during visits, where both researchers will scan the same zone at the same time. Reliability scans will be compared using Cohen’s kappa (acceptable value ≥ 0.6) and percentage agreement (acceptable value ≥ 80%) for categorical variables, and intraclass correlation coefficients (acceptable value ≥ 0.8) for continuous variables. For each reliability scan timestamp, the final data will be an average of both observers’ data.

##### Proportion of OSHC care sessions OSHC staff demonstrate physical activity enabling behaviour

Staff behaviour will be collected at each time point using the System for Observing Staff Promotion of Activity and Nutrition (SOSPAN) direct observation tool.^27^ This tool is specifically designed to be used as an adjunct to SOPLAY. After each SOPLAY visual scan, a second scan is performed documenting the presence and behaviour of staff in relation to physical activity (categorised as either engaged, instructing, promoting [enabling behaviours] discouraging, withholding, punishing [disabling behaviours] or off-task). The SOSPAN has demonstrated strong to excellent validity and inter-observer agreement (75–100% agreement).^27^ Interrater reliability will be captured and analysed as per outcome (1).

##### OSHC service characteristics

Years in operation, number of children enrolled, number of staff, hours of operation, staff qualifications and service governance will be collected at baseline, using survey items we have previously developed.^28^

##### Determinants of implementation behaviour

Determinants of implementation behaviour will be measured at each time point using purpose-designed survey items (mapped onto the TDF framework) that assess various domains relating to implementation of physical activity and screen time in OSHC (knowledge, skills, role and identity, beliefs about capabilities, beliefs about consequences, intentions, environmental context and resources, social influences, and behavioural regulation). OSHC service coordinators will report on their perceived determinants, and we will calculate an average for each distinct domain.

#### Implementation process outcomes (collected from intervention OSHC services only)

A range of implementation process outcomes will be assessed post-intervention to further understand implementation and inform future dissemination efforts:

##### Resource uptake

Server logs from the Activated OSHC website will be used to determine the number of OSHC coordinators and staff who have completed the online training modules.

##### Acceptability, appropriateness, and feasibility

Acceptability, appropriateness, and feasibility will be assessed in the post-intervention survey using Weiner et al’s widely used and validated measures, the Acceptability of Intervention Measure (AIM), Intervention Appropriateness Measure (IAM), and Feasibility of Intervention Measure (FIM).^29^

##### Qualitative interviews

We will interview a subsample of OSHC coordinators and staff in the intervention group (*n* = 30), sampling for maximum variation based on engagement and feedback survey data. Potential participants will be selected evenly from low, medium, and high engagement services and generally negative, neutral, and positive feedback services. A qualitative, semi-structured interview will be used to explore participants’ perceptions and experiences of the intervention program, with specific interview prompts tailored to participant responses to the feedback survey and program engagement. Verbatim transcripts will be generated and thematically coded.^30^ These interviews will identify potential barriers and solutions for widespread implementation and will inform future translation of the Activated OSHC program.

### Sample size and statistical analysis

Based on the intervention increasing the proportion of services meeting the OSHC sector physical activity and screen time guidelines by 20% (from 41% expected in the control group to 61% expected in the intervention group), with 80% power, alpha of 0.05, and 3 repeated measures, we will need 75 OSHC services per study arm. To account for drop-out (which our previous experience of research with OSHC services suggests is rare), we will endeavour to recruit 81 metropolitan services in each study arm (total target sample 162 services). An additional fifteen regional services will be recruited in each study arm (total 30), based on the number of eligible services and anticipated response rate.

Analyses will be performed following the intention to treat principles, with the OSHC service as the unit of analysis. Mixed-effects multivariable logistic regression will be used to determine group-by-time changes in the proportion of intervention and control services meeting the OSHC sector physical activity and screen time guidelines. Group-level changes in children’s physical activity and screen time behaviours, and staff behaviour for enabling physical activity will be assessed using mixed-effects Poisson regression. The models will be adjusted by the stratification factors: state and socioeconomic status tertile. If assumptions warrant it, multiple imputation will be used to replace missing data.

Process outcomes will be analysed using descriptive statistics (e.g., % of services taking up the program, % of intervention services agreeing the program is appropriate). Qualitative interview data will be transcribed and analysed using Thematic Inductive analyses.^31^

### Economic evaluation

A comprehensive cost-effectiveness analysis will be undertaken at the level of the OSHC service. To undertake this the resources used in developing and implementing the intervention will be meticulously quantified. Resources recorded will include the staff time, cost of materials and equipment, travel, and any training sessions. These costs will be assigned monetary values using established unit-cost data from reputable published sources (e.g. Australian Bureau of Statistics Wage Price Index),^32^ or market-rates. Prices will be updated to a common year prior to analysis to account for the effects of inflation.^32^ The total costs to provide the intervention will be calculated individually for all participating OSHC services. The primary outcome of the cost-effectiveness will be presented as an Incremental Cost-Effectiveness Ratio (ICER). The ICER will represent the incremental cost for each additional percentage point improvement in the number of OSHC services adhering to the physical activity and screen time guidelines at 12-month follow-up (i.e. the incremental cost of achieving better adherence in the intervention group versus the control group. Sensitivity analyses will be conducted to assess the robustness of our findings. Both one-way and multi-way sensitivity analyses will be employed to explore the effects of potential variations in key variables like resource utilization, the effectiveness of the intervention, and unit costs on the estimated ICER and therefore cost-effectiveness of the intervention.^33^

## Discussion

This Type 2 hybrid effectiveness-implementation randomised controlled trial aims to implement and critically evaluate the Activated OSHC program’s impact on children’s physical activity and screen time in Australian OSHC services. By rigorously assessing the program’s effect on adherence to newly established physical activity and screen time guidelines, as well as analysing cost-effectiveness, acceptability, and feasibility, the study addresses a significant gap in current extended care practice. The trial’s findings will contribute to the growing body of evidence supporting structured interventions within the childcare sector, especially in out-of-school settings that are crucial for children’s daily activity patterns.

A key strength of this study lies in its hybrid trial design, which intertwines the evaluation of both the effectiveness of the Activated OSHC program and the processes underlying its implementation. This dual focus is complemented by the use of validated observational tools and robust data analysis methods, enabling high reliability and validity of the results. The trial’s design also ensures diverse representation of OSHC services in metropolitan and regional locations across 3 Australian states and, by including a stratified sample based on socioeconomic status, enhances the generalisability of the findings. Additionally, the use of a multidisciplinary approach, incorporating perspectives from stakeholders across the health and education sectors, further underscores the robustness and relevance of the research.

The implications of this study are multifaceted. At its core, the Activated OSHC program is designed to be a sustainable program to enhance current extended care practices. It dovetails with established policy-based accreditation systems (such as SunSmart), providing a dual benefit: promoting healthier behaviours in children and offering OSHC services a mark of distinction through accreditation. Such a credential can serve as a mark of quality, potentially aiding services in positioning themselves favourably in a competitive market.

As we contemplate scaling the program nationally, insights from this trial will be crucial for understanding the nuances of large-scale implementation. The promise of the Activated OSHC program extends to its potential influence on policy, particularly if the findings confirm that the guidelines are effective and sustainable when implemented broadly.

Finally, the national scale of this Australian OSHC physical activity and screen time guidelines and this trial may provide a compelling model for the international community. This could inspire similar initiatives worldwide, showing that strategically designed, policy-aligned interventions can become integral to enhancing the developmental environments of children across diverse settings.

## Supplementary information

Supplementary File 1
