# Supplementary material for: Improving physical activity and screen time in Australian Outside School Hours Care: Study protocol
Source: Pediatr Res. 2024 Aug 23;97(5):1516–21. doi: 10.1038/s41390-024-03464-1 (PMC12119330; doi:10.1038/s41390-024-03464-1)
Supplement: Supplementary file 1 [file 41390_2024_3464_MOESM1_ESM.pdf]

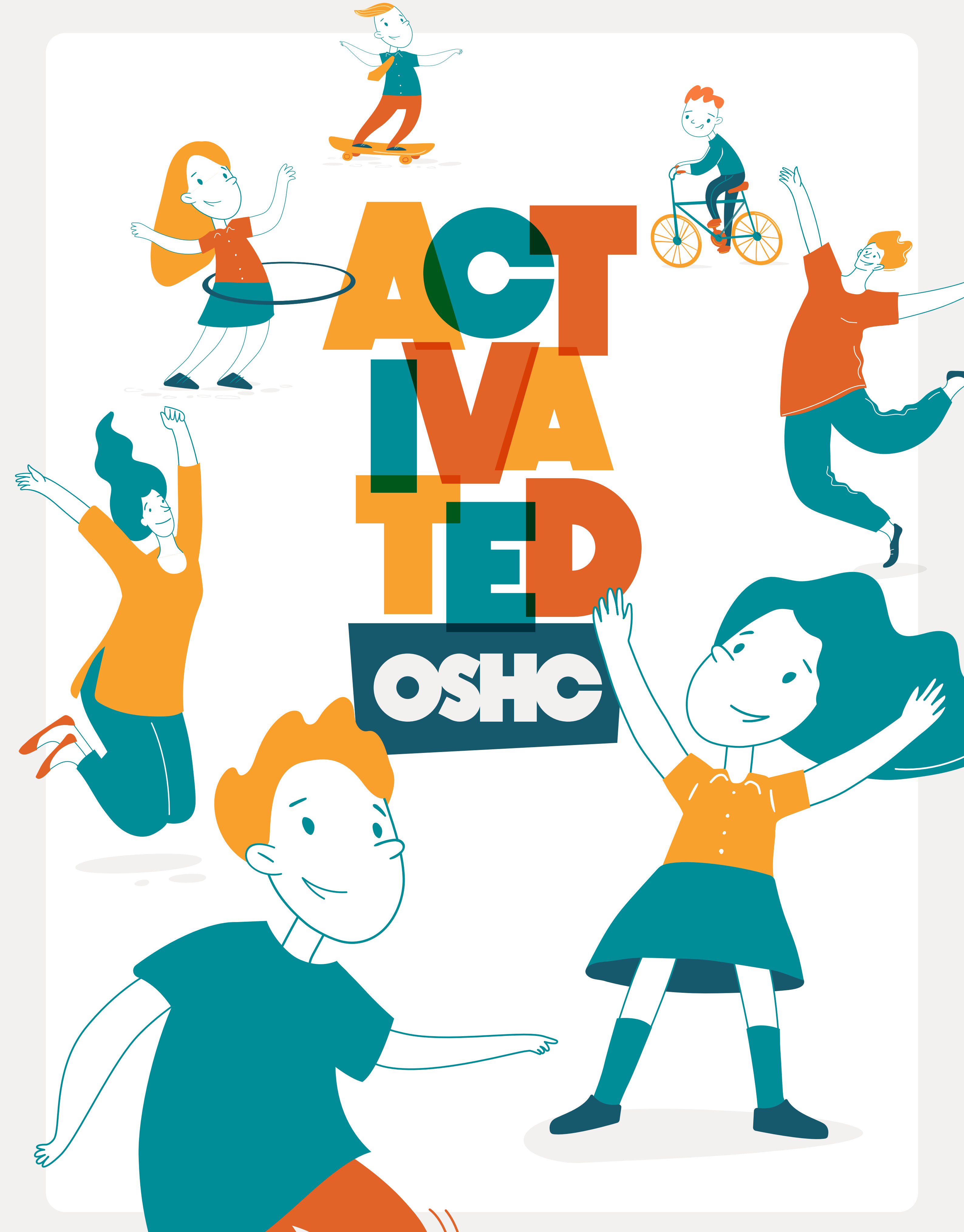

Becoming An Activated OSHC

Version 1.0 February 2022

Contents

Introduction ... 3
Becoming an Activated OSHC ... 3
Why is it important for children to be physically active? ... 4
Why is it important to limit recreational screen time? ... 5
How can OSHC help? ... 6
OSHC physical activity and screen time guidelines ... 7
So what is being done about it? ... 7
Physical Activity Recommendations ... 8
Screen Time Recommendations ... 9
Recommendations for Educators ... 10
Steps to Developing a Physical Activity and Screen Time Policy ... 11
Step 1: Form a working group ... 11
Step 2: Complete Training ... 11
Step 3: Review current Physical Activity Practices ... 11
Step 4: Write and review your Activated OSHC policy ... 11
Step 5: Submit the policy ... 11
Step 6: Implement the policy ... 11
Step 7: Review and update policy as appropriate ... 11
Activated OSHC Checklist ... 12
Sample Physical Activity and Screen Time Policy for OSHC and Vacation Care ... 13
Site ... 14
Rationale ... 14
Objectives ... 14
Implementation times ... 14
1. Scheduling of physical activity and screen time ... 15
2. Environments for physical activity ... 15
3. Equipment for physical activity and screen time ... 15
4. Types of physical activity and screen time ... 16
5. Educator role modelling for physical activity and screen time ... 16
6. Education for physical activity and screen time ... 16
Legislation and National Quality Standards ... 17
Legislation ... 17
National Quality Standards ... 17
Policy Review ... 17
Resources ... 18
Glossary ... 19
References ... 20

Introduction

Becoming an activated OSHC

Activated OSHC is an exciting new initiative to help you improve physical activity and screen time scheduling in OSHC.By becoming an Activated OSHC we will help you understand the new OSHC physical activity and screen time guidelines to enhance your OSHC programming. It allows services to build skills in running facilitated games, make changes and adjustments to physical activity and screen time scheduling and provides an avenue for educator development. Further to that, it helps you to develop a policy for your OSHC service to meet these guidelines as part of everyday practice for children in your service to be happier and healthier.

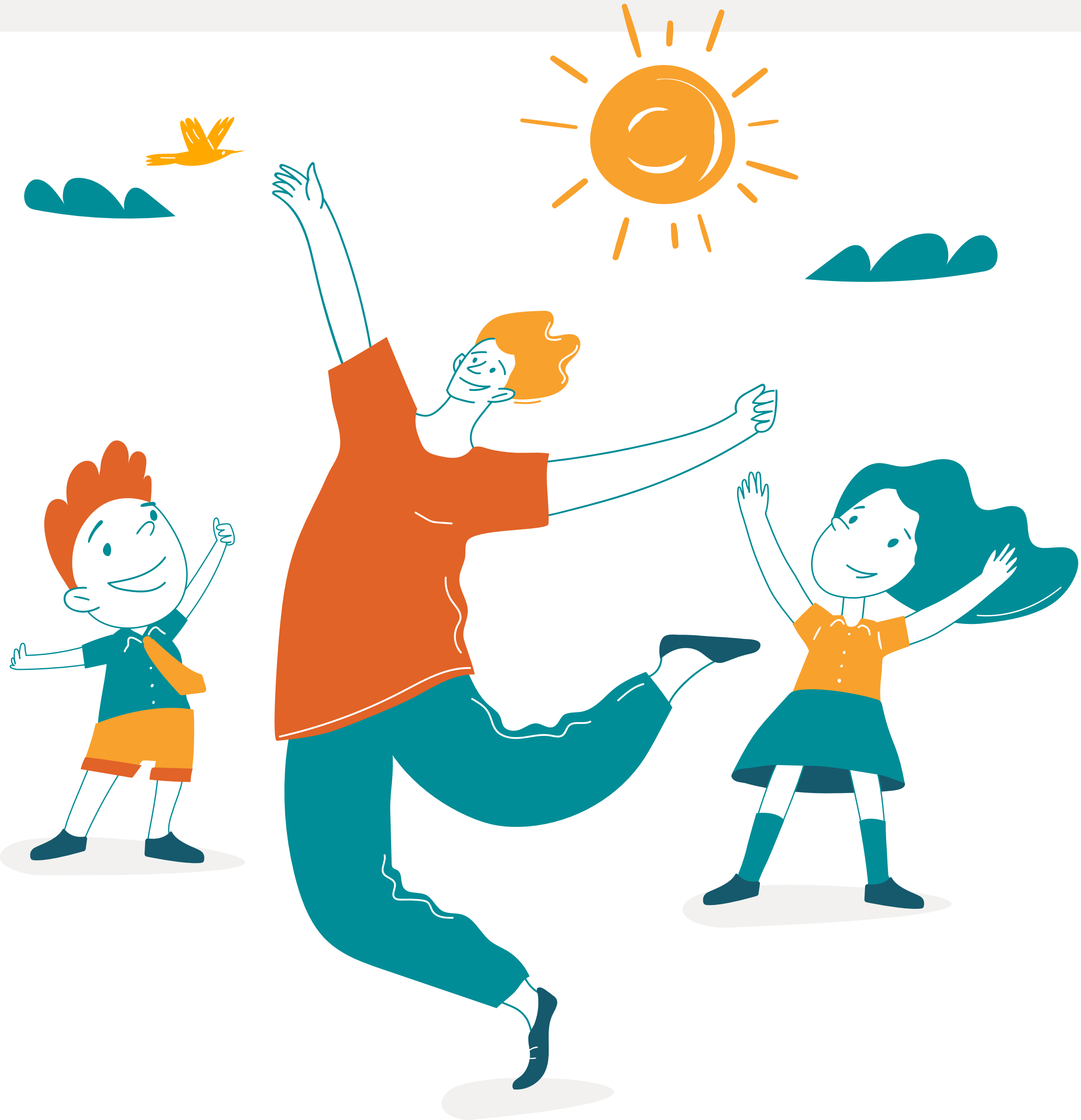

Why is it important for children to be physically active?

Physical activity provides many related benefits for children, including improved heart health, healthy weight, academic performance, sleep, and mental health. Most importantly though, it is fun and part of what being a child should be.

The Australian Government 24-hour movement guidelines state that for children to attain optimal health and well-being they should engage in at least 60 minutes of huff and puff activity.[1]

Most children do not achieve this amount of activity in a day. Recent estimates from 2018 suggest that only 20% of Australian children engage in this much active play each day.[2]

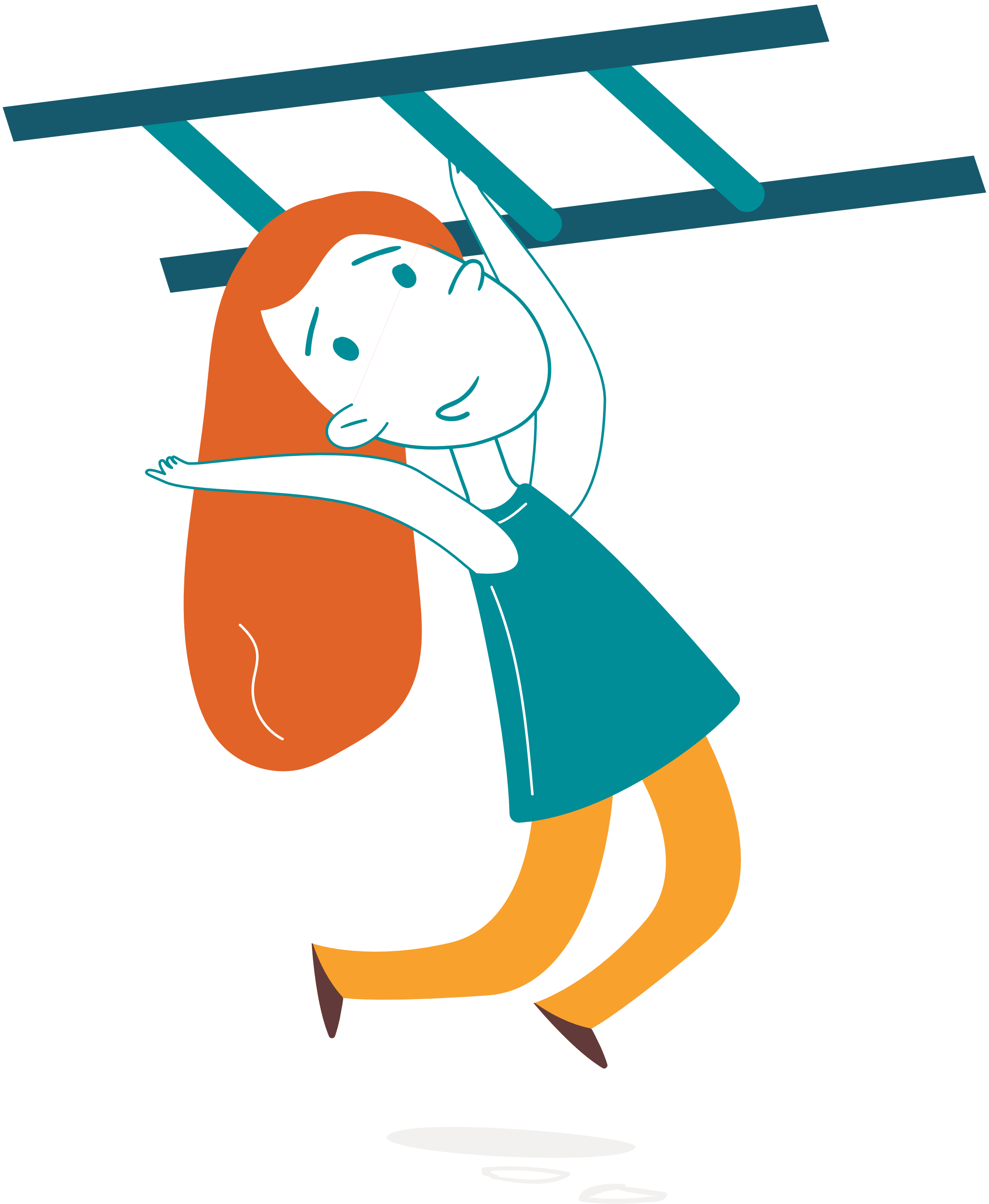

Why is it important to limit recreational screen time?

Recreational screen time refers to using screens for enjoyment, such as watching TV, playing video games, or scrolling through social media. It does not include screen activities for educational purposes (e.g., homework or learning).

The Government guidelines recommend no more than 2 hours of sedentary recreational screen time.1 Excessive recreational screen time is linked with the development of an unhealthy weight, poor heart health, poor physical fitness, poorer academic achievement, low self-esteem, and behavioural difficulties in school aged children.[3]

Only 30% of Australian children meet the screen time recommendation.[2]

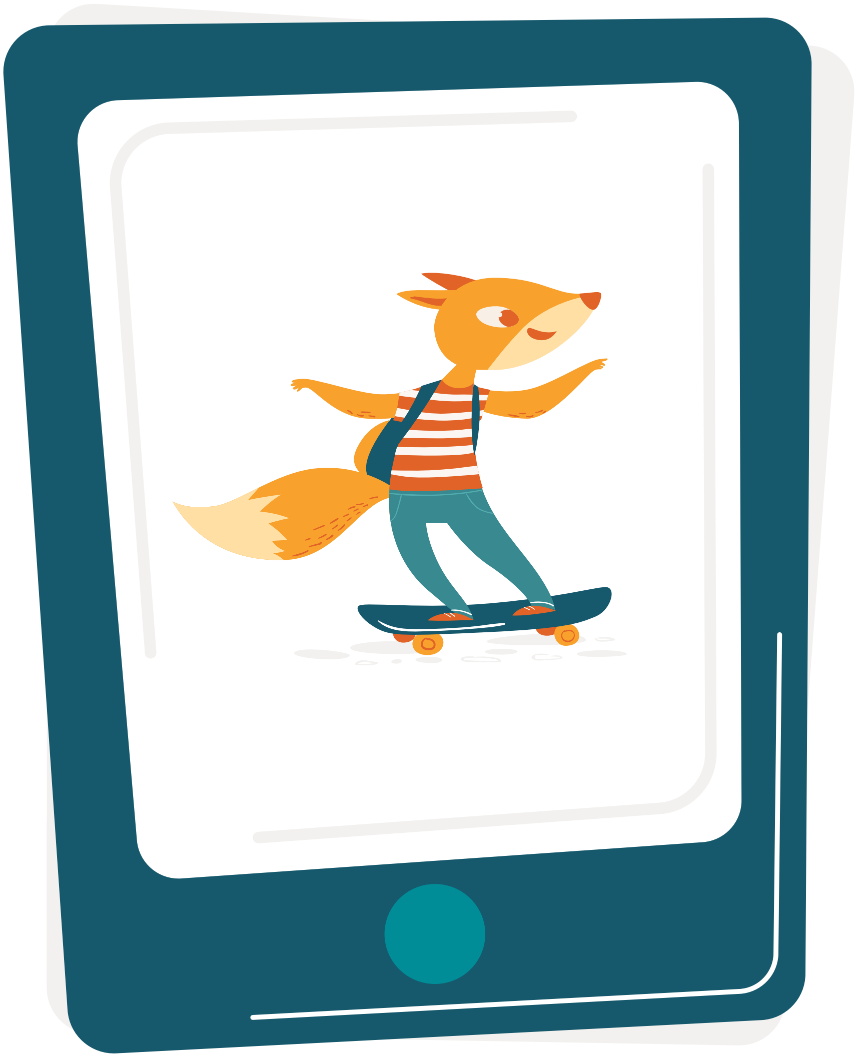

How can OSHC help?

OSHC provides a unique opportunity for children to engage in healthy behaviours. It occurs during a child's "free time". Up to 30% of a child's daily active play occurs during the hours of OSHC (before and after school)[4].

OSHC is an ideal setting, and well placed, to support children to participate in healthy levels of physical activity and screen time. This is a key component of the National Quality Standards – particularly Standard 2.1 *"Each child's health and physical activity is supported and promoted"*.

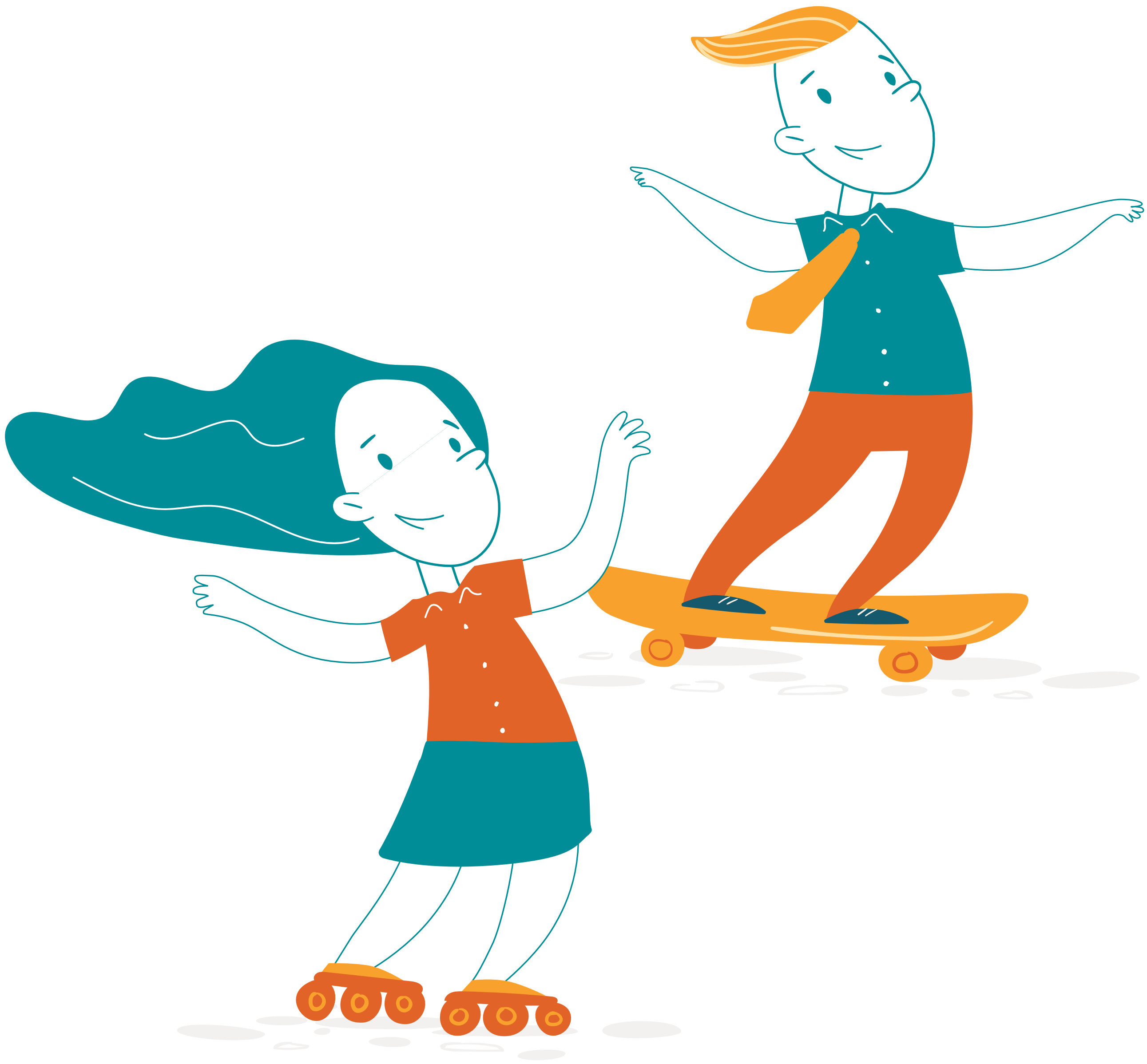

OSHC physical activity and screen time guidelines

So, what is being done about it?

Recently, end-users, stakeholders, and researchers co-developed OSHC Physical Activity and Screen Time Guidelines. These guidelines help OSHC services schedule opportunities for active play and limit screen time access during before school care, after school care and vacation care sessions.

The guidelines below provide recommendations on how to schedule sufficient time so that children can engage in healthy behaviours. They are based on a 3:1 ratio of time schedule: time targeted to be physically active. For example, it has been shown on average children are physically active for around one third of the time they play (because sometimes they are sitting, taking turns, etc). So this means if 90 minutes of physical activity is offered, children on average will be active for 30 minutes of this.

Figure 1: Snapshot of the OSHC physical activity and screen time guidelines.[4]

Session	Physical Activity	Screen Time*
Before School Care	Schedule **45 minutes** of time for children to engage in a variety of physical activites including energetic play. **More is better.**	Discourage children from engaging with screens, with total screen time **no more than 30 minutes. Less is better.**
After School Care	Schedule **90 minutes (1.5 hours)** for children to engage in a variety of physical activity, including energetic play. **More is better.**	Discourage children from engaging with screens, with total screen time no more than **60 minutes. Less is better.**
Vacation Care	Throughout the day schedule at least **2-3 hours of time** for children to engage in a variety of physical activity, including energetic play. **More is better.**	Discourage children from engaging with screens. **No more than 2 hours** over the entire day**. Less is better.**

*Does not include computer use for homework

Physical Activity Recommendations

The following strategies can help children achieve the recommended amount of physical activity at OSHC. Physical activity should be fun and provide opportunities for children of different ages and abilities.

- Free play usually involves a range of sedentary, light, and energetic activities. For children to achieve the recommended amount of energetic physical activity each day, more time scheduled for active play will be needed (e.g., for children to reach 30 minutes of energetic physical activity, approx. 90 minutes of play time or more will need to be scheduled).
- Outdoor free play is an important source of children's physical activity. OSHC services are encouraged to consider ways to offer outdoor play for as much of the OSHC session as possible.
- Indoor spaces for energetic play are important during inclement (hot or wet) weather, and to increase the variety of activities available to children. OSHC services are encouraged to consider ways to offer energetic indoor play opportunities.
- Equipment and music can used to encourage active play (e.g., bats, balls, nature play, construction materials and dancing music).
- Educator-led activities (e.g., playground games and sports) both structured and unstructured may be offered in addition to free play. Games that allow children to be active most of the time (e.g., tag) are preferable to games that involve sitting out or extended waiting for a turn (e.g., tunnel ball).
- Children should be allowed to self-regulate and move freely between energetic play and sedentary activities as they choose.
- Scheduling opportunities for active play, whether indoors or outdoors, each day will help achieve physical activity recommendations.

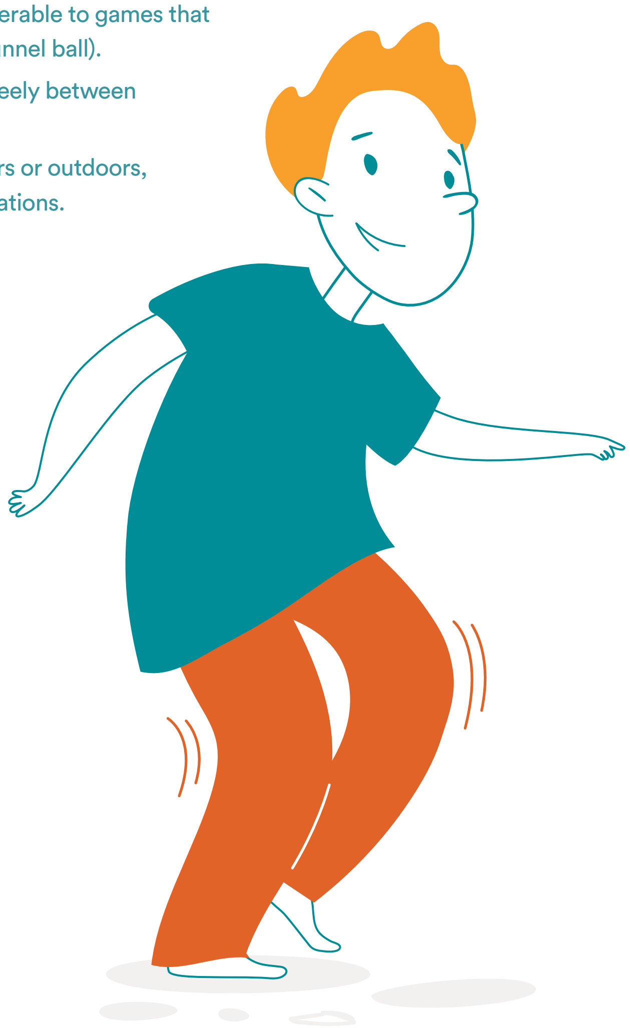

Screen Time Recommendations

The following strategies can help children achieve the recommended amount of sedentary recreational screen time at OSHC. Children should be able to move freely between active play and quiet activities at OSHC. Quiet activities are important for children to relax, feel emotionally settled, and to learn.

- Children need the opportunity to engage in activities other than recreational screen time; an engaging program offered to children in OSHC services is a good way to minimise boredom, challenging behaviours and a desire for recreational screen time.
- Active play, and quiet non-screen based activities (e.g., reading, talking, board games, arts, and crafts) should be offered in preference to recreational screen time.
- Many children, if offered recreational screen time concurrently with other types of activities, will choose screen time. Therefore, it is recommended that recreational screen time is only offered sparingly e.g., only offered at specific times on days (e.g., a rotating day per week/last day of term) or during inclement weather.
- If recreational screen time is offered daily, the time should be restricted and given low priority compared with other activities (e.g., it could be made available during the last 30 minutes of the after school care session).
- Children should be discouraged from accessing personal electronic devices at OSHC, unless for educational purposes.
- OSHC Educators and older children should be encouraged to role model appropriate recreational screen time use (e.g., don't use personal electronic devices during OSHC sessions).

Note: Using electronic devices to complete homework is not recreational screen time. The recommendations above apply to recreational screen activities such as watching TV and DVDs, playing on a computer and electronic games.

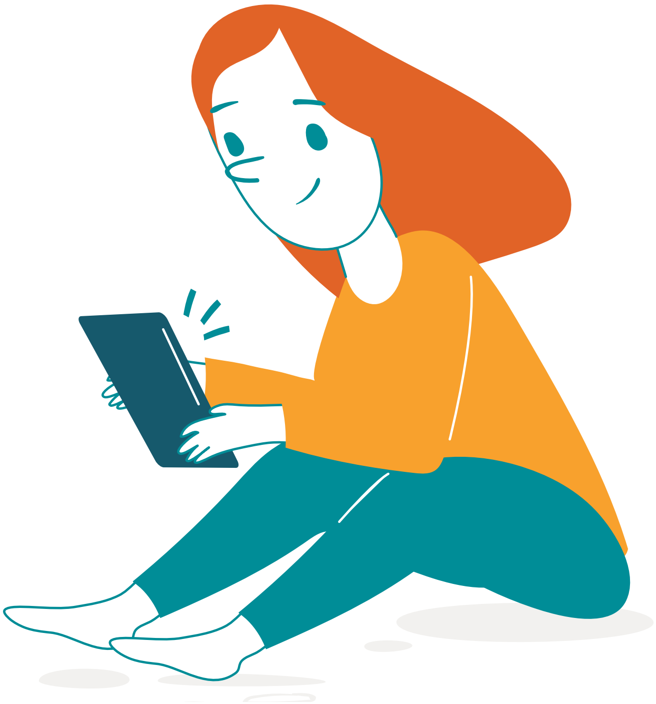

Recommendations for Educators

OSHC educators play a critical role in planning and delivering OSHC programs, including setting and changing the service's culture around physical activity and recreational screen time practices.

It is important that educators understand the importance of physical activity and screen time for children's health and wellbeing and facilitate and model appropriate behaviours. The following educator-focused strategies may assist children in their care to achieve the recommended amount of physical activity and recreational screen time at OSHC.

- OSHC educators have access to regular professional development which provides opportunities to develop their knowledge and skills to help children achieve healthy active behaviours e.g., through short online training modules.
- It is recommended that the OSHC Physical Activity and Screen Time Guidelines be embedded in orientation and annual performance plans and reviews of educators to ensure awareness, understanding and implementation of the guidelines.
- It is recommended that the OSHC Physical Activity and Screen Time Guidelines be embedded into OSHC policies and procedures; and any other documents reflecting how your OSHC service operates (e.g., your service philosophy).
- When planning the OSHC program, attention should be given to prioritising physical activity over recreational screen time every day. This is particularly important for children who may only attend the service one or two days per week. Efforts should be made to meet time specific physical activity and screen time recommendations every day.
- Educators should be encouraged to role model healthy physical activity and screen time behaviours by engaging children during active play (e.g., actively supervising and engaging in play activities and offering verbal encouragement) and minimising the use of personal electronic devices.
- Ongoing training to develop OSHC educators' skills to facilitate active games is encouraged to help make it easier to implement the guidelines.

Steps to developing a Physical Activity and Screen Time Policy

A policy will help achieve the OSHC physical activity and screen time guidelines. The following steps are recommended:

Step 1: Form an Activation Team

The role of the Activation team is to develop and review the physical activity and screen time policy for your OSHC. The policy will give your service a roadmap to ensure you are providing high quality active play and limited recreational screen time every day. The Activation team is made up of an **Activated OSHC Champion** and **Activated OSHC Supporters** (all OSHC educators).

Step 2: Complete Training

The **Activated OSHC Champion** is either the coordinator or a senior educator within your service. They are responsible for making sure all educators in the service complete the online training relating to implementing the OSHC Guidelines and submitting the policy to the **Activated OSHC Website**.

Step 3: Review current physical activity practices

You can use the following checklist (page 10) to determine what is already being done in your OSHC service and where you may be able to improve.

Step 4: Write and review your Activated OSHC policy

Now it's time to write your **Activated OSHC** policy. It should include statements on how the service will implement the physical activity and screen time guidelines. We've provided a sample policy which you can choose to use or adapt for your service. This is a great opportunity to include children in planning your service's activities.

Step 5: Submit the policy

Once you have completed your policy, you're ready to submit it! Please submit via the **Activated OSHC Website**. This will be reviewed by the **Activated OSHC Accreditation team**. You will be provided with feedback. Once assessed and approved your service will be an **Activated OSHC** and you will receive signage and access to the FREE ONLINE resources.

Step 6: Implement the policy

Put it into action! Make sure that all educators and families using your service are aware that you are an **Activated OSHC**. Print off the **free online resources** to hang posters in your service and distribute the parent resources.

Step 7: Review and update policy as appropriate

The policy will only work if your OSHC users and educators are familiar with it. Routinely promote being an **Activated OSHC** by:

- including in your educator handbook / orientation information
- have all new educators complete online training
- including **ACTIVATED OSHC** as a set agenda item on meetings at appropriate times (e.g., twice a year, including first staff meeting of the year)
- including information in your enrolment packs
- using newsletters to promote your physical activity and screen time policy

Ways to review the effectiveness of your policy may be:

- surveying educator and families
- surveying children – what new activities are you doing
- brief audits of use of screen activities/outdoor play areas/equipment for active play

Remember your ACTIVATED OSHC accreditation lasts for 3 years, so plan how to maintain and then renew your accreditation

Activated OSHC Checklist

Physical activity scheduling

- ☐ Time is allocated daily for active, free play and/or educator led play
- ☐ 45 minutes for physical activity opportunities scheduled during before school care
- ☐ 90 minutes for physical activity opportunities scheduled during after school care
- ☐ 2 or more hours per day allocated during vacation care (if your service offers vacation care)

Screen time scheduling

- ☐ Recreational screen activities are not available at the same time as physical activity opportunities
- ☐ If recreational screen time is scheduled, it is limited and given lower priority
- ☐ If provided, less than 30 minutes per day for the entire before school care session
- ☐ If provided, less than 60 minutes per day for the entire after school care session
- ☐ If provided, less than 2 hours in total for the entire vacation care session (if applicable)

Environment for physical activity and screen time

- ☐ Outdoor play spaces available for all OSHC sessions including open and undercover areas
- ☐ Indoor energetic play space available
- ☐ Equipment for Physical Activity and Screen Time
- ☐ Access to various sports equipment such as: balls, skipping ropes, cones, goals, climbing
- ☐ Access to sidewalk games e.g., hopscotch/four square/snakes and ladders
- ☐ Children do not bring personal electronic devices to OSHC for recreational purposes*

Educator involvement and role modelling

- ☐ Educator led sports and/or energetic games are offered in addition to free play
- ☐ Educator encourage children towards energetic play
- ☐ OSHC educators' model healthy behaviours e.g., active with children in games, not accessing personal electronic devices at work

Online training

- ☐ All OSHC educators to complete the online training modules for Activated OSHC
- ☐ Policy Promotion – promote Activated OSHC policy to both educators and families
- ☐ Provide new and current educators with a copy of Activated OSHC policy and access to online training modules at staff meetings and/or via email.
- ☐ Provide OSHC families information about Activated OSHC policy and practices
- ☐ Use Activated OSHC materials in service for children to see

Policy Review

- ☐ The OSHC reviews it Activated OSHC policy every 3 years to ensure information is relevant and up to date

*Devices used for homework excepted e.g., laptop

Sample Physical Activity and Screen Time Policy for OSHC and Vacation Care

OSHC services are welcome to use this policy in full or adapt it. Please ensure it accurately reflects your service.

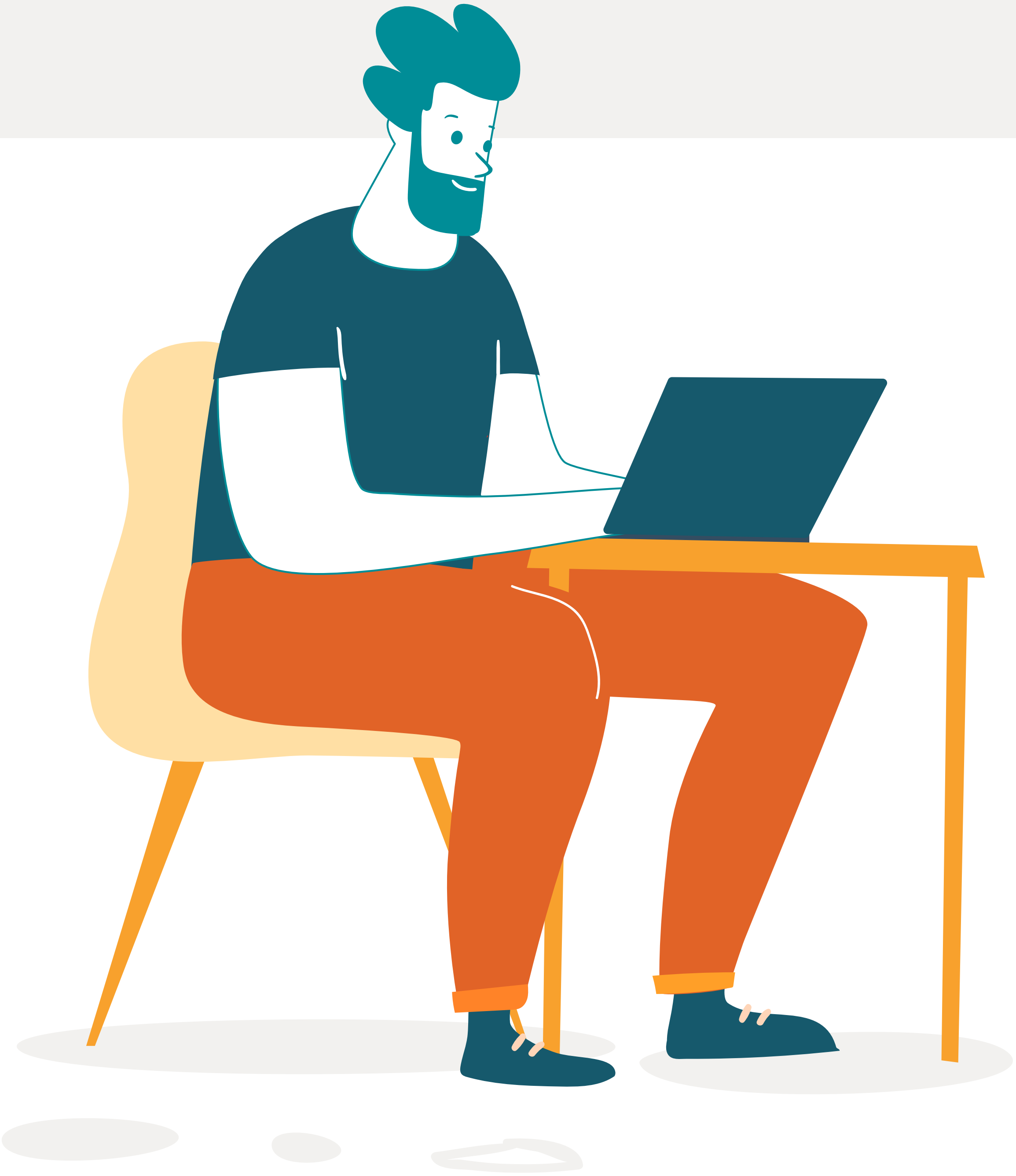

Physical Activity and Screen Time Policy for OSHC and Vacation Care

Site

This policy applies to all OSHC services, on and off-site.

Rationale

Physical activity is essential for children to grow, learn and develop optimally. Benefits include better cognitive and academic outcomes, and improved heart health, fitness, sleeping patterns, and mental wellbeing.

Screen time is associated with poorer physical, social, and intellectual development, though offered in moderation, can have some benefits. Unfortunately, however, most Australian children get too much screen time.

OSHC (including Vacation Care) plays a vital role in children's physical activity and screen time. Children who attend OSHC spend a considerable part of their day there and generally attend regularly, so the activities they do at OSHC substantially influence their ongoing daily activity patterns and lifestyle. Therefore, by having a service-wide policy that ensures a healthy balance of physical activity and screen time, OSHC services can positively impact the health and wellbeing of many children.

Objectives

This policy has been developed to:

- Encourage children to achieve their recommended daily amount of physical activity and screen time every day in OSHC and Vacation Care
- Work towards having both outdoor and indoor environments that provide ample opportunities for children to be active
- Assist OSHC educators to model and facilitate healthy physical activity and screen time behaviours.
- Ensure families, children and new educators are informed of the service's physical activity and screen time policy.

Implementation times

Every day: The policy will be implemented daily in before school care, after school care, and vacation care, within reason.

Exceptions: The policy may not be implemented in exceptional circumstances, such as inclement weather (e.g., wet weather or extreme heat). In addition, it may occasionally not be implemented, for example, on the last day of term. However, exceptions should not be made every week (e.g., it is not acceptable to disregard the policy every Friday).

1. Scheduling of physical activity and screen time:

Note that many OSHC and Vacation Care services are now choosing not to offer recreational screen time daily, because they recognise that children are likely to be getting plenty of recreational screen time outside of OSHC/Vacation Care. Therefore, two sample statements are provided – you should select the statement that best fits your OSHC values.

☐ **Children will not be offered recreational screen time at OSHC**

☐ **Children will be offered energetic outdoor (and indoor) play between the following hours:**

- Before school session: from **[start time]** to **[end time]** - totalling at least 45 minutes for energetic play, preferably more, and preferably later in the session, when most children are in attendance.
- After school session: from **[start time]** to **[end time]** - totalling at least 90 minutes for energetic play, preferably more, and preferably earlier in the session, when most children are in attendance.
- Vacation care: from **[start time]** to **[end time]** and **[start time]** to **[end time]** - totalling at least 2-3 hours for energetic play, preferably more.
- Children will be offered recreational screen time between the following hours (note: screen time for homework is not recreational screen time, so does not count towards these limits):
- Before school session: from **[start time]** to **[end time]** - no more than 30 minutes, preferably less or none, and minimising scheduling screen time opportunity at the same time as physical activity.
- After school session: from **[start time]** to **[end time]** - no more than 60 minutes, preferably less or none, and minimising scheduling screen time opportunity at the same time as physical activity.
- Vacation care: from **[start time]** to **[end time]** - no more than 2 hours, preferably less or none, and minimising scheduling screen time opportunity at the same time as physical activity.

*Note that if individual recreational screen time activities are offered (e.g., video games and iPads) and per child time limits are used (e.g., 10 minutes per child) the TOTAL time the devices are offered must still meet the above limits.

2. Environments for physical activity:

Children will have access to a range of energetic/active play spaces:
(e.g., oval, playground equipment, sandpit, basketball courts, list all that apply).

Work towards having indoor and outdoor spaces available for energetic play, so active play continues to be possible even in inclement weather.

3. Equipment for physical activity and screen time:

Children will be offered access to a range of sports equipment:
(e.g., balls, trikes, cones, list all that apply).

Work towards having energising music playing in active play zones to encourage children to dance and create a lively atmosphere.

If offering recreational screen time activities, work towards providing screen time activities that promote social interaction and movement (e.g., active video games such as the Wii) rather than handheld devices (e.g., iPads).

4. Types of physical activity and screen time:

Outdoor free play (unstructured, child-initiated activity) is the main physical activity in OSHC and Vacation Care. It is offered every day.

Educator-led play (where OSHC educators lead a group of children in an activity, such as a sport or game) may also be offered. When adult-led games are offered, games that maximise children's active participation will be used (e.g., avoid games that involve long waits for a turn, or games that involve sitting out).

[If recreational screen activities are offered] Recreational screen time may include watching television for rest, videogames, and handheld devices. When television is used, educational content will be favoured. When videogames are offered, work towards offering games that maximise children's active participation (e.g., active video games such as "Wii Fit" and "Let's Dance" and avoid games that involve long waits for a turn). Avoid using recreational screen time as a reward.

Children will not use mobile phones or personal electronic devices for games/browsing at OSHC and Vacation Care.

5. Educator role modelling for physical activity and screen time:

Educators will role model positive physical activity and screen time behaviour, including:

- Standing and actively moving around when supervising outdoor free play.
- Encouraging and positively reinforcing children's physical activity.
- When on duty, using personal mobile phones for emergencies only, not for browsing.
- At times, joining in with games.

6. Education for physical activity and screen time:

Physical activity is incorporated into educational programs and activities, supporting children's learning, development, and wellbeing.

Healthy physical activity and screen time are reinforced through displays (e.g., posters). (include all strategies provided at your OSHC/Vacation Care)

Families and educators are provided with healthy physical activity and screen time information through newsletters, brochures, noticeboards, and the service's website. (include all strategies provided at your OSHC/Vacation Care)

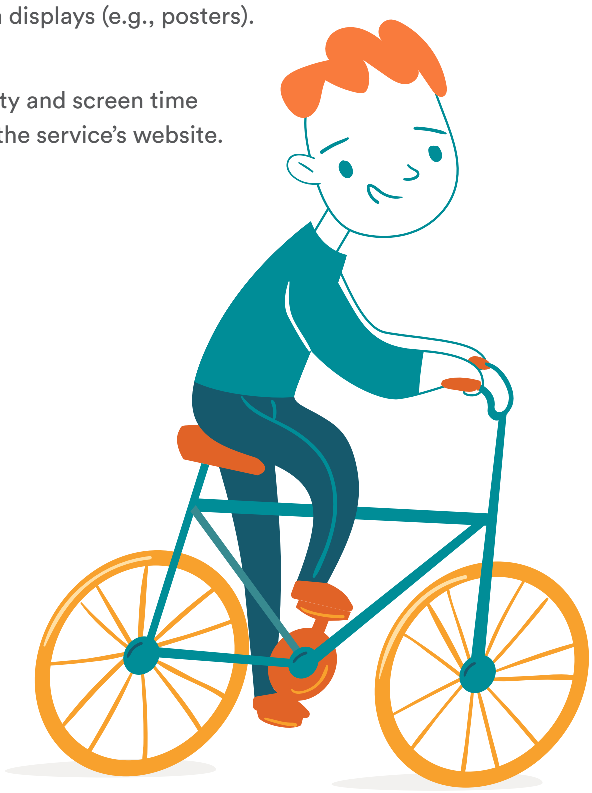

Legislation and National Quality Standards

Legislation

By creating and following this policy, supports us to meet the following components of the Education and Care Services National Law Act 2010 (Version No0.13)

- Part 6 – Operating an Education and Care Services
- Item 167: Offence relating to protection of children from harm and hazards
- Item 168: Offence relating to required programs

National Quality Standard

This physical activity and screen time policy relates directly to:

- Quality Area 2: Children’s Health and Safety

And links to:

- Quality Area 3: Physical Environment
- Quality Area 5: Relationships with Children
- Quality Area 6: Collaborative partnerships with families and communities
- Quality Area 7: Governance and Leadership

This policy and how it is implemented may be used as supporting evidence for how we are meeting the National Quality Standard for these quality areas during our next quality rating and assessment.

Policy Review

Management and OSHC educators will regularly review the effectiveness of the physical activity and screen time policy (at least twice per year) and update as necessary. Including children in the review process is encouraged.

Date of next policy review:

ACTIVATED OSHC accreditation Renewal

Management and OSHC educators will ensure that their Activated OSHC accreditation remains current. Renewal is required every 3 years.

Date of Activated OSHC Accreditation Renewal:

Resources

Activated OSHC Website

https://www.activatedoshc.com.au

Queensland children's Activity Network

https://www.panosh.com.au

Playing for Life - SportAUS

Fun and interactive games to develop children's skills, confidence, and lifelong interest in sport. Playing for Life activity cards and video demonstrations are designed for everyday use by teachers, coaches and OSHC educators and parents.

https://www.sportaus.gov.au/p4l

Heart foundation: Eat Smart, Play Smart Manual

https://www.healthykids.nsw.gov.au/downloads/file/teacherschildcare/EatSmartPlaySmart_Manual_ThirdEdition-V7.pdf

Clearinghouse for Sport

https://www.clearinghouseforsport.gov.au/australian-sport-publication-archive/australian-sports-commission/asc_programs/asc_programs_-_aasc/aasc-playing-for-life

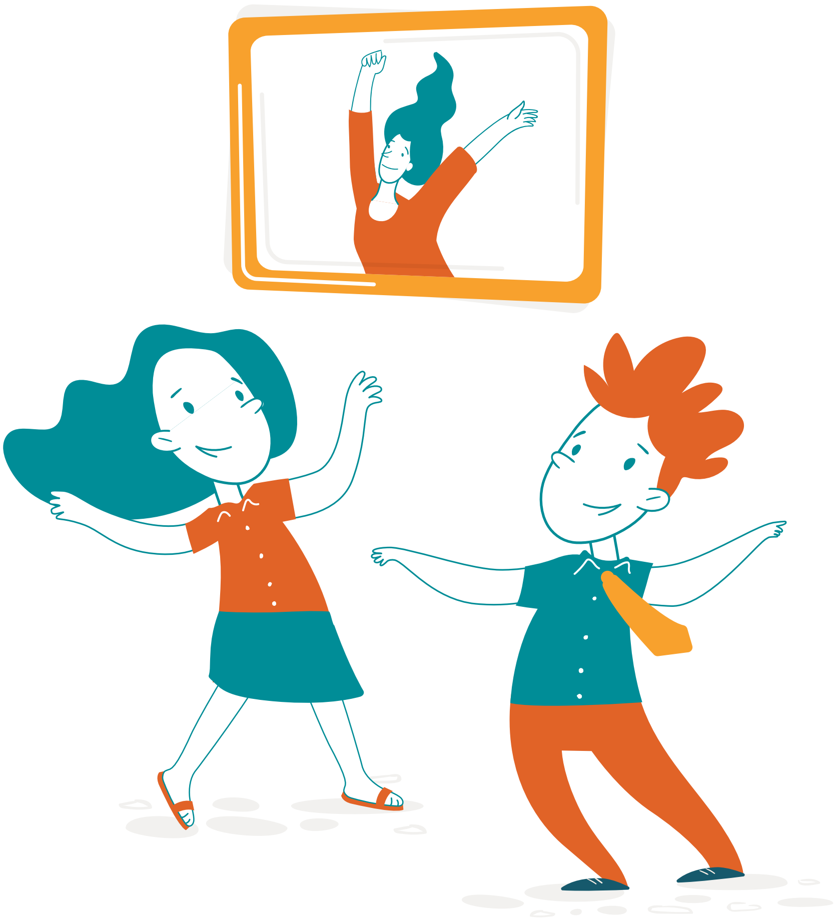

Glossary

Physical Activity

Any bodily movement produced by skeletal muscles that require energy expenditure e.g., running, walking, active recreation, play and/or sports.

Recreational Screen Time

Time spent using devices/screens such as a computer, television, gaming console, personal electronic device (mobile phone/tablet) that does not promote activity and is for enjoyment.

Sedentary Behaviour/Activity

Aany waking behaviour that requires minimal energy expenditure while is a sitting, reclining, or lying posture.

Active Play/Energetic Play

Any activity that involves moderate to vigorous burst of high energy play i.e., makes them huff and puff.

Free Play

A type of play where children have full freedom to engage in any type of play they want. During free play children can express themselves in the way they choose.

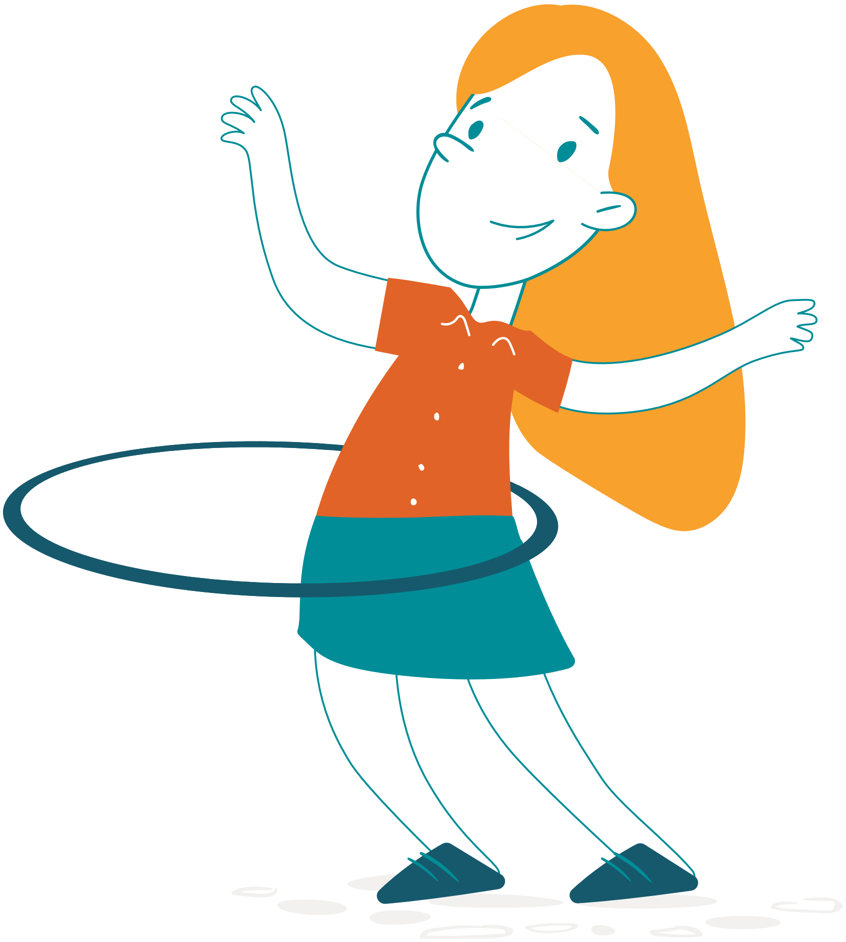

References

1. **Okely AD OT, Jones R, Cliff D, Parrish A, Loughran S et al**
Australian 24-Hour Movement Guidelines for Children (5-12 years) and Young People (13-17 years): An Integration of Physical Activity, Sedentary Behaviour, and Sleep In: Department of Health, editor. Canberra: Australian Government; 2019

2. **Schranz N, Glennon V, Evans J, Gomersall S, Hardy L, Hesketh KD, et al**
Results from Australia's 2018 report card on physical activity for children and youth. Journal of physical activity and health. 2018;15(s2): S315-S7

3. **Saunders TJ, Vallance JK**
Screen time and health indicators among children and youth: current evidence, limitations, and future directions. Applied health economics and health policy. 2017;15(3):323-31

4. **Virgara R, Phillips A, Lewis L, Richardson M, Maher C**
Development of Australian physical activity and screen time guidelines for outside school hours care: an international Delphi study. International journal of behavioural nutrition and physical activity. 2021;18(1):1-14

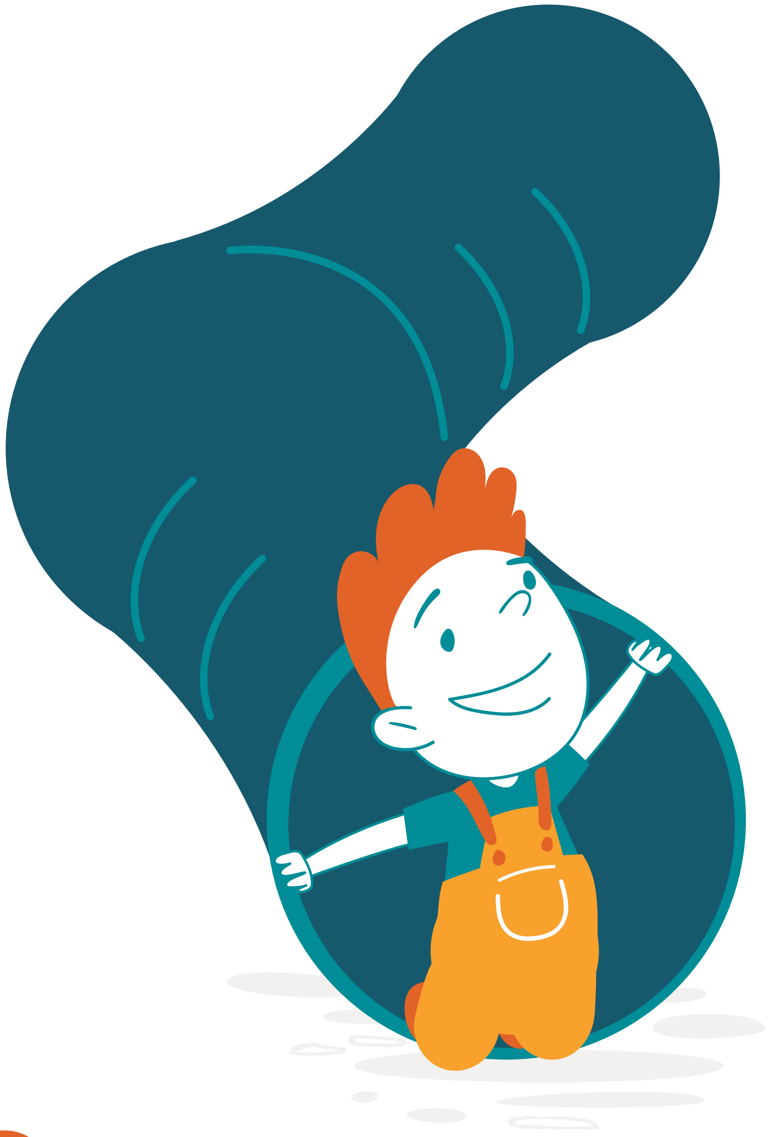

ACTIVATED

OSHC

©Adtivated OSHC 2022
